# The association of *CYP11A1* gene polymorphisms with the polycystic ovary syndrome patients

**DOI:** 10.1590/1806-9282.20231293

**Published:** 2024-07-19

**Authors:** Sihad Salim Hakeem Alyousif, Burcu Ozbakir, Ali Cenk Ozay, Pinar Tulay

**Affiliations:** 1 Near East University, Faculty of Medicine, Department of Medical Genetics – Nicosia, Cyprus.; 2 Near East University, Faculty of Medicine, Department of Obstetrics and Gynecology – Nicosia, Cyprus.; 3 Near East University, DESAM Research Institute – Nicosia, Cyprus.; 4 Cyprus International University, Department of Obstetrics and Gynecology – Nicosia, Cyprus.; 5 Near East University, Center of Excellence, Genetics and Cancer Diagnosis-Research Center – Nicosia, Cyprus.

**Keywords:** PCOS, Polymorphism, CYP11A1, SNP

## Abstract

**OBJECTIVE:**

The objective of this study was to investigate the allele frequencies of polymorphisms in genes *CYP11A1* rs4886595 and *CYP11A1* rs4887139 that are responsible for the steroidogenesis mechanism in polycystic ovary syndrome patients and control females.

**METHODS:**

Samples were obtained from the Department of Obstetrics and Gynecology in the Near East University Hospital from September 2019 to December 2019. Only the nonobese patients between the ages of 18–40 years were included in this study following informed consent. Obese patients and patients more than 40 years of age were excluded from the study. Nonobese women and normal ovulation were included in the control group. DNA was isolated from blood samples. Real-time polymerase chain reaction (PCR) was used to analyze single nucleotide polymorphisms (SNPs) in various genes linked to polycystic ovary syndrome. The studies were carried out using the samples obtained from 120 women, of whom 55 were nonobese and had normal ovulation, and 65 were polycystic ovary syndrome patients. The allelic frequencies of SNPs in genes linked to polycystic ovary syndrome were calculated using real-time PCR outcomes.

**RESULTS:**

The variation of the *CYP11A1* rs4887139 G>A did not show any significance, while the variation of *CYP11A1* rs4886595 C>A showed significant differences between the patient and the control groups (p=0.01), respectively.

**CONCLUSION:**

Future research ought to focus on elucidating the susceptible causes of polycystic ovary syndrome with a wide range of SNPs and more sample size. The genome-wide association studies in polycystic ovary syndrome patients of different origin will be important to identify candidate genes as well as proteins that are implied in polycystic ovary syndrome risk.

## INTRODUCTION

Polycystic ovary syndrome (PCOS) is a complicated and common issue that affects 5–20% of women of reproductive age. PCOS has been diagnosed in around 105 million women aged approximately 15–49 years worldwide^1^. PCOS continues to be one of the most difficult medical conditions to treat because of its intricate nature, characteristics of development, and effects on women's lives from the teenage years until the postmenopausal phase^2^. The most common outcomes of PCOS are female anovulation, marked by hyper-androgen and insulin resistance, and it is among the most common sources of irregularities in menstruation, amenorrhea, and oligo-menorrhea, which is the most common reason for female infertility^3^. PCOS is marked by gonadotropin deficiencies, elevated androgen levels, insulin resistance, chronic anovulation, and irregular menstrual cycle. Reproductive anomalies are the habitual feature of PCOS. Hormones play a dominant role in the ovary's function and the menstrual cycle's management, which preserves fertility. If there is a chronic hormone level disruption in females, it can disrupt the activity of the ovary, contributing to the development of a cyst within the ovary, whereas androgen has risen in females affected by PCOS beyond its normal range^4^. Pregnant women with PCOS are more likely to undergo pregnancy difficulties and unfavorable outcomes in their offspring, which may be associated with factors related to the pathology of the syndrome and its related comorbidities^5^. Furthermore, there is an elevated risk of developing metabolic disorder, cardiovascular diseases, and type II diabetes. The diagnosis of PCOS includes the presence of a biochemical and/or clinical androgen surplus, polycystic ovarian morphology (PCOM) on ultrasound, and anovulation or oligo-ovulation^6^. Diabetes mellitus is 5–10 times more common in PCOS women^7^. The genetic background is suspected in approximately 79% of PCOS patients, while the environment and lifestyle account for 21%^8^.

Polycystic ovary syndrome is a multigenic disease in which the heterogeneous, physiological, and biochemical phenotype is associated with both genetic and environmental influences. Approximately 40% of degree-relatives of PCOS females are also expected to develop PCOS compared with women in the general population (with an incidence of 4–6%)^9^. Several genes are predicted to have a contribution to PCOS etiology, and the recent analyses of the genome-wide studies have discovered a number of candidate genes^10^. Epigenetic and environmental influences, such as an unhealthy diet or low to absence of physical exercise, probably complicate any underlying genetic predisposition^11^. It has been suggested that metabolic syndrome in PCOS is a progressive condition; however, there are limited confirming follow-up data to support this theory. Over a 20-year period, 193 patients had follow-up visits every 5 years as part of a research conducted by Carmina et al. Although the patients’ oligo/anovulation and hyperandrogenism improved, metabolic problems continued, particularly in relation to an increase in abdomen circumference. Therefore, fat tissue could be involved in PCOS's metabolic problems. A recent study has established that the fourth decade of life is associated with a decrease in PCOS characteristics^12^. Moreover, it is thought that problems with carbohydrate metabolism and a chronic inflammatory process associated with PCOS might impede the contact between the mother and the embryo and trophoblast invasion, which may result in miscarriage^13^.

The *CYP11A1* (cytochrome P-450 11A1) gene belongs to the cytochrome P450, family 11. This gene is located on chromosome 15q23-q24. *CYP11A1* regulates the synthesis of pregnenolone from cholesterol in the inner membrane of the human mitochondria is one of the most promising candidate genes for PCOS enzyme codes, which cultivate the first and the most important step in the production of steroid hormones^14^.

The objective of this study was to investigate the allele frequencies of polymorphisms in genes *CYP11A1* rs4886595 and *CYP11A1* rs4887139 that are involved in the steroidogenesis mechanism in PCOS patients and control females. We hypothesize that the allelic frequencies of single nucleotide polymorphisms (SNPs) would be different in the two groups.

## METHODS

### Ethical approval

Ethical approval was granted by the Near East University, Health Sciences Ethical Committee (YDU/2019/67-784).

### Sampling and processing

Whole blood samples were obtained from September to December of 2019 from 120 patients undergoing routine checkup at the Department of Obstetrics and Gynecology in the Near East University Hospital. Each patient signed an informed consent form. The patients’ clinical information was obtained. The inclusion criteria for the study included nonobese women between the ages of 18 and 40 years. The exclusion criteria for the study were obese women and nonobese women of more than 40 years of age. Samples were divided into two groups for analysis. Nonobese women with normal ovulation and considered not to have PCOS were included in the control group, and non-obese PCOS patients were included in the patient group. The study group included a total of 120 women, of whom 55 were nonobese and had normal ovulation, and 65 were PCOS patients.

### DNA extraction and real-time PCR

DNA was extracted from each sample following the manufacturer's protocol (Invitrogen pure link genomic DNA mini kit, USA). NanoDrop (Nanodrop ND200, Thermo Scientific, Pittsburgh, USA) was used to measure the concentration and purity of the DNA at the 260:280 ratio. Allelic frequencies at specific SNP locations within the genes *CYP11A1* rs4886595 and *CYP11A1* rs4887139 were determined using real-time polymerase chain reaction (PCR) (LightCycler 480 Sybr Green, Roche Life Science) following the manufacturer's protocol. A final concentration of 25 μM forward and reverse primers was used in the reaction mixture. Each reaction contained 2 μL of DNA. All of the PCRs were performed in a laminar flow hood to avoid contamination. The high-resolution melting method analysis was used to investigate the allelic frequencies of the two SNPs within *CYP11A1*, and the thermal cycler software was used to calculate the cycle of threshold (Ct) and melting temperature (Tm) values. The PCR conditions for amplification were followed at 56°C annealing for 30 s according to the manufacturer's instructions.

Statistical Packages for the Social Sciences (SPSS version 10, Chicago, USA) were used in this study. Descriptive statistics and Mann-Whitney test were applied. Both Kolmogorov-Smirnov test of normality and the Mann-Whitney U test were implemented where applicable because the data did not support parametric assumptions. A p-value of 0.05 was chosen as the degree of significance.

## RESULTS

The heterozygosity status of the SNPs was investigated using the real-time PCR analysis by evaluating the cycle of threshold (Ct) for each amplification. The whole number of cycles required for the fluorescent signal to cross the threshold is shown by the Ct values. Melting temperature (Tm) values were also recorded for each amplification. When the DNA is 50% double-stranded and 50% single-stranded, Tm shows the melt curve. In HRM analysis, following PCR amplification, the amplicons produced melted gradually. This allows fluorescence to be emitted, which is detected using real-time PCR equipment. Due to the variances in Tm values, these melt curves have various shapes.

The results showed that, for the PCOS group, *CYP11A1* rs4887139 polymorphism, higher percentage of patients were found to be homozygous (89.2%), while the heterozygotes (3.0%) were found in low percentage of the patients (Table 1). Among the control group for the *CYP11A1* rs4887139 polymorphism, the results show a higher percentage (90.9%) of patients who were homozygous, while the heterozygous was found in a lower percentage of the patients of about 3.6%.

**Table 1:** The percentage of *CYP11A1* rs4887139 and *CYP11A1* rs4886595 heterozygosity in the polycystic ovary syndrome and control groups.

CYP11A1 rs4887139	Number of samples in patients	Percentage	Number of samples in the control group	Percentage
Homozygous	58	89.2	50	90.9
Heterozygous	2	3	2	3.6
Total	60	92.3	52	94.6
No result	5	7.7	3	5.4
Total	65	100	55	100
CYP11A1 rs4886595				
Homozygous	45	69.2	42	76.4
Heterozygous	14	21.5	10	18.2
Total	59	90.7	52	94.6
No result	6	9.3	3	5.4
Total	65	100	55	100

The homozygosity of *CYP11A1* rs4486595 was high of about 69.2%, while the heterozygosity was low of about 21.5%. Referred to the control group for the *CYP11A1* rs4886595 polymorphism, a higher percentage (76.4%) of patients were found to be homozygous, while the heterozygous was found to be in a lower percentage of about 18.2% (Table 1).

Mann-Whitney U test was conducted on the results (Table 2) to investigate the differences between homozygosity and heterozygosity of the patients using the melting temperature (Tm) analysis. The *CYP11A1* rs4487139 Tm in the patient group showed that the heterozygosity was significantly higher than the homozygosity (mean±SD; 90.70±0.28; mean±SD; 88.29±0.95; p<0.05, respectively). In contrast, Tm of *CYP11A1* rs4487139 for the control group showed that the heterozygosity was significantly higher than the mean homozygosity (91.50±1.97; 88.15±1.07; p<0.05, respectively).

**Table 2:** Descriptive statistics for heterozygosity evaluation of *CYP11A1* rs4887139 using Tm values in the patient and control groups.

Patient group						Control group						p-value between the patient and control groups
	Mean	SD	Median	Min	Max		Mean	SD	Median	Min	Max	
Homozygous	88.29	0.95	88.8	86.0	89.30	Homozygous	88.15	1.07	88.70	86.20	90.70	p=0.203
Heterozygous	90.70	0.28	90.70	90.50	90.70	Heterozygous	91.50	1.97	91.50	90.10	92.90	

Mann-Whitney U test was implemented (Table 3) to investigate the difference between homozygosity and heterozygosity in the patient group using the melting temperature (Tm) of *CYP11A1* rs4886595 gene polymorphism. The Tm of *CYP11A1* rs4886595 for the patient group showed that the heterozygosity was significantly higher than the mean homozygosity (87.80±1.39; 86.04±1.41; p<0.05), respectively. While the Tm of *CYP11A1* rs4486595 for the control group showed the heterozygosity was significantly higher than the mean homozygosity (87.34±2.49) (85.38±1.59) (p<0.05), respectively.

**Table 3:** Descriptive statistics for heterozygosity evaluation of *CYP11A1* rs4886595 using Tm values in the patient and control groups.

Patient group						Control group						p-values between the patient and control groups
	Mean	SD	Median	Min	Max		Mean	SD	Median	Min	Max	
Homozygous	86.04	1.41	86.10	80.10	89.60	Homozygous	85.38	1.59	85.95	82.10	88.60	p=0.01
Heterozygous	87.80	1.39	87.70	84.60	90.30	Heterozygous	87.34	2.49	87.60	82.70	90.60	

## DISCUSSION

The results of this study showed that the patient and control groups have no significant difference in the Tm of *CYP11A1* rs4887139 (p=0.203), while the mean±SD of Tm in the patients group was remarkably higher than the mean±SD of Tm in the control group in *CYP11A1* rs4886595 (p=0.01), respectively. However, when further analysis was done, it was shown that there was a significant difference in the Tm rs4886595 among the patient and control groups, while no significant difference was observed for the Tm of rs4887139.

The ovary is considered a major organ in the female reproductive system, and its interruption due to endocrine anomalies may lead to female infertility. PCOS is a metabolic and hormonal disorder that devastates women throughout their reproductive years^15^. Nonetheless, regarding its heterogeneity and complex structure, PCOS remains unclear in commonly recognized clinical significance. Moreover, the existence of PCOM in these patients happens to be usual occurrences in most PCOS patients^16^. Approximately 95% of females with this syndrome have decreased degree of follicle-stimulating hormonal and polycystic ovaries at the initial follicular stage, which prompt antral-follicle development and elevate LH expression^17^. In this study, two SNPs of the *CYP11A1* (rs4887139 and rs4886595 variants) were chosen to examine the possible association with the disorder. The global allelic frequency of *CYP11A1* rs4887139 is 0.8902, whereas the global allelic frequency of *CYP11A1* is 0.8291. Neither of the variants are considered pathogenic since neither had been reported in ClinVar. These variants are located in the noncoding regions of the gene. The chemical transformation of cholesterol to pregnenolone that is facilitated by the cytochrome enzyme (P450scc) is the most important step in the biosynthesis of steroid hormones in the ovary. Genetic alterations in the regulatory region of *CYP11A1* and *CYP17A1* genes are associated with the pathogenesis of PCOS. Common polymorphisms of *CYP17A1* and *CYP11A1* genes are hypothesized to predict the individual's susceptibility to PCOS^18^. Similar studies were performed previously in such seven SNPs within *CYP11A1* (rs12917295, rs11632698, rs1484215, rs6495096, rs4887139, rs9806234, and rs4886595) in PCOS patients that were genotyped. The results show that the genotype "GG" of rs4887139 was associated with a high PCOS risk with odds ratio (OR)=1.79, 95% CI=1.04–3.10, p=0.035, and the genotype "CC" of rs4886595 with increased PCOS risk with OR=4.29, 95% CI=0.90–20.36, p=0.04^19^. Similar results were also reported previously in such genotypic distributions of the SNP *CYP11A1* rs4077582 in PCOS patients that were substantially different from the controls in 106 Egyptian females between the ages of 18 and 45 years. Thus, they concluded that *CYP111A1* rs4077582 is associated with the pathophysiology of PCOS, implying that *CYP11A1* rs4077582 may alter the P450scc compound activity, and, as a result, androgen production^20^. In contrast, another study showed insignificant differences in Tm among the patient and control groups in rs4887139^21^.

This study has a number of limitations, one of which was the sample size. The small sample size in both the case and control groups might impair the reproducibility of the findings. Although a few research studies aimed to investigate the heterozygosity at these two polymorphisms (rs4887139 G>A and rs4886595 C>A), further analysis is required to establish the correlation between these polymorphisms and PCOS. Another limitation was the number of SNPs investigated. Future studies should include more SNP genotyping.

## CONCLUSION

In this study, the differences in the heterozygosity status of the alleles at the rs4886595 C>A within *CYP11A1* involved in PCOS recorded significant differences in the patient and control groups, while the heterozygosity of rs4887139 G>A within *CYP11A1* was shown to be insignificant in both groups. Therefore, future research ought to focus on elucidating the susceptible causes of PCOS with a wide range of SNPs and more sample size. More genome-wide association studies in PCOS patients of different origin will be important to recognize prospective genes as well as proteins that are implied in PCOS risk.
